# Extracranial Stereotactic Body Radiotherapy in Oligometastatic or Oligoprogressive Breast Cancer

**DOI:** 10.3389/fonc.2020.00987

**Published:** 2020-06-26

**Authors:** Fabian Weykamp, Laila König, Katharina Seidensaal, Tobias Forster, Philipp Hoegen, Sati Akbaba, Stephan Mende, Stefan E. Welte, Thomas M. Deutsch, Andreas Schneeweiss, Jürgen Debus, Juliane Hörner-Rieber

**Affiliations:** ^1^Department of Radiation Oncology, Heidelberg University Hospital, Heidelberg, Germany; ^2^Heidelberg Institute of Radiation Oncology (HIRO), Heidelberg, Germany; ^3^National Center for Tumor Diseases (NCT), Heidelberg, Germany; ^4^Department of Obstetrics and Gynecology, Heidelberg University Hospital, Heidelberg, Germany; ^5^Department of Radiation Oncology, Heidelberg Ion-Beam Therapy Center (HIT), Heidelberg University Hospital, Heidelberg, Germany; ^6^Clinical Cooperation Unit Radiation Oncology, German Cancer Research Center (DKFZ), Heidelberg, Germany; ^7^German Cancer Consortium (DKTK), Partner Site Heidelberg, Heidelberg, Germany

**Keywords:** oligometastatic, oligoprogression, stereotactic body radiotherapy (SBRT), breast cancer, local control, progression free survival, distant control, overall survival

## Abstract

**Purpose/Objective:** Oligometastatic disease (OMD) and oligoprogressive disease (OPD) describe tumor states with a limited metastasization. In contrast to other disease states, treatment of OMD or OPD has not yet become common for breast cancer. We sought to understand the outcomes and toxicities of this treatment paradigm.

**Material/Methods:** We retrospectively analyzed female breast cancer patients with OMD (≤3 metastases) or OPD (1 progressive lesion) who received stereotactic body radiotherapy (SBRT) for their respective extracranial metastatic lesions between 01/2002 and 07/2019. Survival analysis was performed using the Kaplan-Meier method with log-rank test being used for evaluation of significance. Cox regression was used to detect prognostic outcome factors. Toxicity was evaluated using the Common Terminology Criteria for Adverse Events (CTCAE v. 5.0).

**Results:** Forty-six patients (70% OMD; 30% OPD) with 58 lesions met criteria for inclusion. The majority of treatments (34 out of 58; 58.6%) were delivered from 2017 to 2018. Treatment sites were bone, liver, lung [*n* = 19 (33%) for each site], and adrenal gland [*n* = 1 (1%)]. Median biologically effective dose (BED at α/β = 10) was 81.6 Gy (range: 45–112.5 Gy) and median planning target volume was 36.60 mL (range: 3.76–311.00 mL). At 2 years, local control (LC) was 89%, distant control (DC) was 44%, progression free survival (PFS) was 17% and overall survival (OS) was 62%. Multivariate analysis identified the diagnosis of a solitary metastasis as an independent prognostic factor for superior DC (HR = 0.186, CI [0.055; 0.626], *p* = 0.007) and PFS (HR = 0.363, CI [0.152; 0.863], *p* = 0.022). OS was independently inferior for patients treated at a higher age (HR = 5.788, CI [1.077; 31.119] *p* = 0.041). Nine (15.5%) grade I° and one (1.7%) grade II° toxicities were recorded, with no grade III° or higher toxicities.

**Conclusion:** Extracranial SBRT in breast cancer patients with OMD or OPD was well-tolerated with excellent LC. SBRT should especially be offered to younger OMD and OPD breast cancer patients with only one metastasis. The increase in utilization since 2017 points toward a growing acceptance of SBRT for OMD and OPD in breast cancer.

## Background and Purpose

The concept of oligometastatic disease (OMD) was first described by Weichselbaum and Hellman during the 1990s (1). Up to 10% of patients with metastatic breast cancer are thought to belong to this category (2). Recent studies defined OMD as a maximum of five present metastases (3–5). A few years after the initial description of OMD, surgical metastasectomy emerged as a promising treatment modality (6). A non-invasive alternative to treat limited metastases is stereotactic body radiotherapy (SBRT), which has been proven effective and well-tolerated during the last decade (7–10). SBRT allows to deliver high ablative radiation doses, while sparing surrounding normal tissue. Two recently published randomized controlled Phase-II trials, one of them including 20% breast cancer patients (SABR-COMET trial), could demonstrate, that local therapy of metastases in patients with OMD leads to a prolonged progression free survival (PFS) and even increases overall survival (OS) (11, 12). Moreover, Wong et al. demonstrated in a study with a similar design (61 patients; 12% breast cancer histology), that breast cancer histology was the strongest positive prognostic factor for local control (LC), PFS and OS (13). It was already shown during the first pilot studies in this field, that breast cancer patients benefit significantly better from ablative radiation of their oligometastases than any other primary tumor (14).

On the contrary, the concept of oligoprogressive disease (OPD) describes a widespread tumor stage, where usually up to five metastases are progressive after systemic therapy. In times of emerging targeted therapies and immunotherapies the concept of OPD gains importance as few resistant subclones leading to progression of solitary metastases are observed more frequently (15). OMD and OPD are not well-established as disease concepts for breast cancer patients, in contrast to other tumor entities. On the contrary, the recent 8th edition of the TNM classification of lung cancer describes a M-subgroup for patients with OMD (16). There is no such subclassification in breast cancer patients with OMD (17). This is a surprising fact, considering a 10 year OS of up to 75% in breast cancer patients with single bone metastases which surmounts the OS of many other tumors, even in their early stage (4). A survey of Canadian medical oncologists revealed that 65% would rather start systemic therapy in breast cancer patients with OMD, than even consider a SBRT at all (18). As SBRT for oligometastatic breast cancer patients is a relatively new disease concept, most studies in this area only include a small number of patients and mostly consist of only one specific (5) or predominant (19–21) location of metastases. Additionally, OPD patients are not represented in these studies.

The aim of the study was therefore to evaluate outcome and prognostic factors following SBRT in oligometastatic and oligoprogressive breast cancer patients.

## Methods

### Patient and Treatment Characteristics

We retrospectively analyzed female breast cancer patients treated with ablative SBRT for their extracranial metastases in the Department of Radiation Oncology at Heidelberg University Hospital from 01/2002 to 07/2019. Patients were excluded from the study if they were not treated with SBRT, but with palliative intent or palliative doses. SBRT was defined as an ablative dose with single fraction doses > 4 Gy and number of fractions < 10.

SBRT was performed if patients were either classified inoperable, technically or medically, or refused surgical resection. At our center, patients with brain metastases are only treated with SBRT for extracranial metastases under special circumstances (e.g., excellent performance status and completed whole brain radiotherapy).

A 4D computed tomography (CT) scan with 3 mm slice thickness was used for treatment planning except for bone metastases. Furthermore, contrast-enhanced CT scans were applied for target delineation in all patients except for the ones who were treated with SBRT for bone metastases. When available, diagnostic magnetic resonance (MR) images or positron emission tomography (PET) scans were additionally used for target volume delineation. For lesions in the lower lung, an abdominal compression device was used. Patients were positioned in an individually shaped vacuum mattress. Number of fractions and single-fraction doses were adjusted to size and location of the metastases. Lung metastases were classified to be peripheral or central according to the RTOG definition (22, 23). Before 2012, lung SBRT was performed with a single fraction of 24–30 Gray (Gy) prescribed to the 90–95% isodose line. From 2012 on, peripheral lung metastases were treated with three fractions of 15–18 Gy, prescribed to the minimum 65% isodose covering at least 95% of the PTV. Central lesions received eight fractions of 7.5 Gy prescribed to the minimum 80% isodose line covering at least 95% of the PTV and very central lesions (<2 cm distance to main bronchus) 10 fractions of 5 Gy to the 95% isodose. The same fractionation schemes were applied to liver and adrenal metastases. Bone metastases received three fractions of 9 Gy, prescribed to the minimum 80% isodose covering at least 95% of the PTV. Before 2012, a single fraction of 24 Gy was used, prescribed to the minimum 80% isodose covering at least 95% of the PTV.

The biologically effective dose (BED) was used to compare treatment schemes with the clinical result. An α/β ratio of 10 Gy was assumed for the metastases. BED was calculated using the linear-quadratic model (24).

$$\begin{array}{l} \text{B}\text{E}\text{D}\left(\text{G}\text{y}\right)\;=\;\text{f}\text{r}\text{a}\text{c}\text{t}\text{i}\text{o}\text{n}\text{a}\text{l}\;\text{d}\text{o}\text{s}\text{e}\\ \;\times \;\text{n}\text{u}\text{m}\text{b}\text{e}\text{r}\;\text{o}\text{f}\;\text{f}\text{r}\text{a}\text{c}\text{t}\text{i}\text{o}\text{n}\text{s }\left(\text{1}+\frac{\text{f}\text{r}\text{a}\text{c}\text{t}\text{i}\text{o}\text{n}\text{a}\text{l}\;\text{d}\text{o}\text{s}\text{e}}{\alpha /\beta }\right) \end{array}$$

### Endpoints and Statistical Methods

LC, distant control (DC), PFS and OS were calculated starting from the last day of SBRT. In this study, LC refers to the high dose area surrounding the irradiated metastases. Recurrences anywhere else where classified as distant failure. LC was calculated based on each lesion. DC, PFS and OS were calculated per patient. Toxicity was evaluated using the Common Terminology Criteria for Adverse Events (CTCAE v. 5.0).

First follow-up was performed 6 to 8 weeks after completion of the SBRT with a clinical examination as well as a contrast fluid CT or MRI scan of the irradiated area. Further follow-up was done according to German guidelines and regularly included a contrast-enhanced CT scan of the thorax/abdomen every 3 months.

LC, DC, PFS and OS were estimated using the Kaplan-Meier method. Survival curves were compared between groups in an univariate analysis applying the log-rank test or cox regression analysis. Multivariate cox models were performed including all variables with p ≤ 0.1 from univariate analysis. A *p* ≤ 0.05 was considered statistically significant. All statistical analyses were performed with SPSS software (IBM SPSS Version 24.0).

This retrospective study was approved by the Ethics committee of the University Hospital Heidelberg (Reference number: S-855/2019).

## Results

Most patients had early stage breast cancer at primary diagnosis (47.8%) and received neoadjuvant or adjuvant chemotherapy (71.7%), mainly anthracycline/cyclophosphamide/taxane based regimes. All patients had a controlled or recently resected primary tumor, with adequate adjuvant radiotherapy of the breast or chest wall according to current national guidelines (25–27). Further patient characteristics are shown in Table 1. Table 2 illustrates patient characteristics at time of the respective SBRT. Median age at time of SBRT was 55 years (range 27–82), with a median time from primary diagnosis to development of metastases of 43.0 months (range 5.4–265.0). The majority of patients had oligometastatic disease (70%), with a maximum of three present and therefore irradiated metastases in this subgroup. Oligoprogressive patients (30%) had had one progressive lesion which was treated with SBRT. Lung, liver and bone were equally represented as SBRT organs (each 33%), with a single case of a metastasis in the adrenal gland (1%). All 14 OPD patients also had stable metastases in further organs, and two OMD patients received SBRT in two different organs. In total, 16 patients (34.8%; Table 2) had metastases in more than one organ. Within 4 weeks prior to SBRT, 27 patients (58.7%) received endocrine therapy, nine patients (19.5%) received anti-Her2/neu treatment and eight patients (17.4%) received chemotherapy (taxan *n* = 3; vinca alkaloid *n* = 2; capecitabine, pegylated liposomal doxorubicin, carboplatin/gemcitabine, each *n* = 1). Within 4 weeks after SBRT, 26 patients (56.5%) received endocrine therapy, nine patients (19.5%) received anti-Her2/neu treatment and seven patients (15.2%) received chemotherapy (vinca alkaloid *n* = 3; capecitabine, pegylated liposomal doxorubicin, carboplatin/gemcitabine, carboplatin, each *n* = 1). No significant difference in terms of acute toxicity was found in patients, who had received chemotherapy prior to or after SBRT (*p* = 0.823). Table 3 describes details of the SBRT treatment and toxicities. Median prescribed total dose was 28 Gy (range 24–60) applied in a median of three fractions (range 1–10) resulting in a median biologically effective dose of 81.6 Gy (range 45.0–112.5 Gy). Overall response rate was 96.6%, with two progressive SBRT lesions (3.4%) in the first follow-up. Nine (15.5%) grade I° toxicities were documented after first follow-up, namely pneumonitis (*n* = 4), reflux esophagitis, abdominal pain, nausea, fatigue and liver edema (each *n* = 1). One (1.7%) grade II° pneumonitis was described. No grade III° or higher toxicities were reported. Toxicity as well as LC, DC, PFS, and OS were not significantly different before the year 2012, when single dose SBRT was used (*p* > 0.05).

**Table 1:** Analysis of patient characteristics at initial diagnosis.

Estrogen positive	35	76.0%
Progesterone positive	29	63.0%
Her2/neu rich	8	20.5% (*n =* 39)
“Triple negative”	3	7.7% (*n =* 39)
Well-differentiated (G1)	2	4.9% (*n =* 41)
Moderately differentiated (G2)	25	61.0% (*n =* 41)
Poorly differentiated (G3)	14	34.1% (*n =* 41)
Histology		
Ductal	15	39.5% (*n =* 38)
Lobular	6	15.8% (*n =* 38)
Ductolobular	1	2.6% (*n =* 38)
Not otherwise specified	16	42.1% (*n =* 38)
UICC stage at initial diagnosis		
Early stage (I–II)	22	47.8%
Locally advanced (III)	9	19.6%
Metastatic disease (IV)	15	32.6%
Initial chemotherapy	33	71.7%
- Neoadjuvant	18	54.5%
- Adjuvant	15	32.6%
- Anthracycline/cyclophosph-amide/taxane based	18	54.5%
- Plus anti Her2/neu agent	7	21.2%
Initial surgery	46	100%
Breast conserving surgery	24	52.2%
Mastectomy	22	47.8%
Axillary dissection	34	73.9%

**Table 2:** Analysis of patient characteristics at time of or after SBRT.

Median age	55 years	Range 27–82 years
Median Karnofsky Score	90%	Range 70–100%
Median time from initial diagnosis to metastasization^*^	43 months	Range 5.4–265.0 months
≤3 metastases in total (=oligometastatic)	32	70.0%
≥3 metastases in total, but 1 progressive (=oligoprogressive)	14	30.0%
Any metastases in other organs	16	34.8%
- Bone	9	56.3%
- Liver	4	25.0%
- Brain	1	6.3%
- Bone, liver, brain	1	6.3%
- Bone, liver, lung, lymphatic	1	6.3%
Chemotherapy within 4 weeks before SBRT	8	17.4%
Chemotherapy within 4 weeks after SBRT	7	15.2%
Number of SBRT lesions		
One	37	80.4%
Two	8	17.4%
Three	1	1.7%

*SBRT, stereotactic body radiotherapy*.

* *excluding patients with synchronous metastasization*.

**Table 3:** Analysis of the SBRT lesions.

Localization of SBRT lesion		
- Bone	19	32.8%
- Lung	19	32.8%
- Liver	19	32.8%
- Adrenal gland	1	2.0%
Histological sample taken from metastasis	3	5.2%
SBRT lesions progressive in planning CT scan	6	10.3%
Median prescribed total dose	28 Gy	Range 24–60 Gy
Median fractions	3	Range 1–10
Median dose inhomogeneity	80%	Range 65–100%
Median EQD2 (α/β = 10)	68.8 Gy	Range 40.0–93.4 Gy
Median BED (α/β = 10)	81.6 Gy	Range 45–112.5 Gy
BED (α/β = 10) >100 Gy	25	43.1%
PTV volume	median 36.6 mL	Range 3.8–311.0 mL
Result in first follow-up		
Complete remission	1	1.7%
Partial remission	21	36.2%
Stable disease	34	58.6%
Progressive disease	2	3.4%
Toxicity CTC I^°^	9	15.5%
Toxicity CTC II^°^	1	1.7%

*BED, biologically effective dose; CTC, Common Terminology Criteria; PTV, planning target volume; SBRT, stereotactic body radiotherapy*.

**Figure 1:**
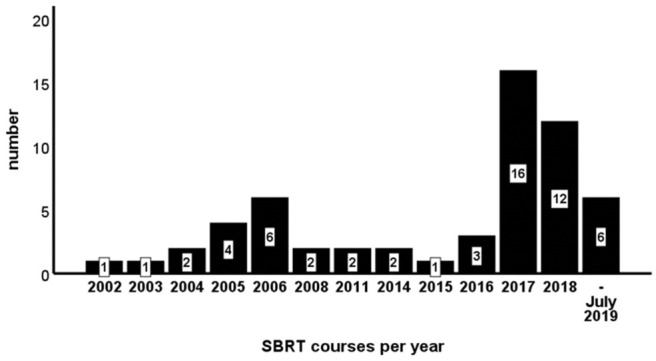
Stereotactic body radiotherapy (SBRT) courses per year.

Median clinical follow-up was 21 months (range 2.4–93.0). During the analyzed period from 01/2002 to 07/2019, the majority of patient (58.6%) was treated recently, beginning in the year 2017 (Figure 1).

### Local Control

**Figure 2:**
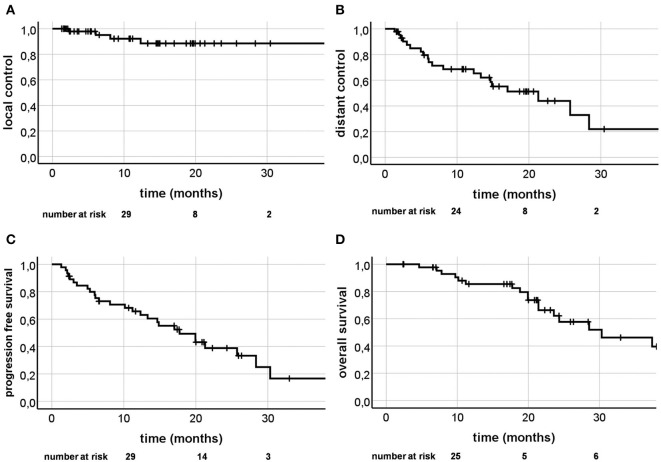
Kaplan-Meier curves; Local control **(A)**, distant control **(B)**, progression free survival **(C)**, and overall survival **(D)**.

**Figure 3:**
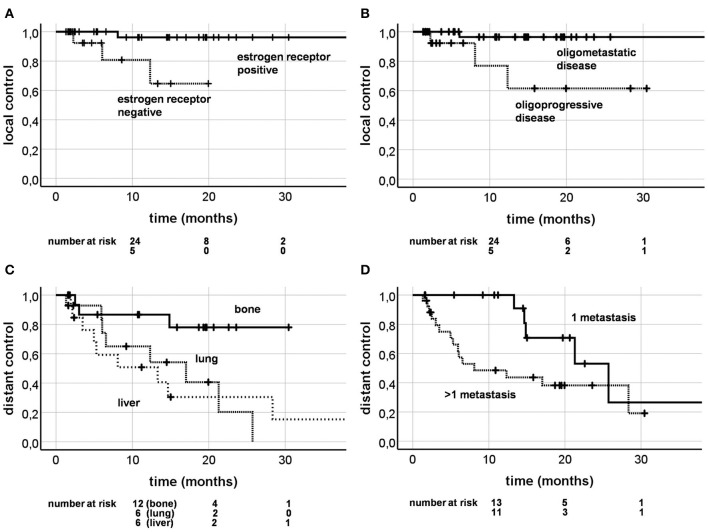
Kaplan-Meier curves; local control depending on estrogen receptor positivity (**A**; *p* = 0.045) and oligoprogressive disease (**B**; *p* = 0.037); distant control depending on the irradiation site (**C**; *p* = 0.018) and present metastases (**D**; *p* = 0.056).

Four out of 58 lesions (6.9%) recurred during follow-up period with 1 and 2 year LC of 92.2% and 88.5% (Figure 2A). Univariate analysis (Table 4) revealed Karnofsky Performance Score (KPS) (HR = 0.840, CI [0.721; 0.977], *p* = 0.024) and estrogen receptor positivity (HR = 0.098, CI [0.010; 0.946], *p* = 0.045; Figure 3A) as positive prognostic factors, whereas OPD was associated with worse local control (HR = 11.234, CI [1.159; 108.877], *p* = 0.037; Figure 3B) as well as chemotherapy 4 weeks before or after SBRT (HR = 14.149, CI [1.461;137.050], *p* = 0.022). After adjusting for potential confounding variables on multivariate analysis, none of the aforementioned variables stayed significant (Table 5).

**Table 4:** Univariate analysis of prognostic factors influencing LC, DC, PFS, and OS.

	**LC**			**DC**			**PFS**			**OS**		
**Factors**	**HR**	**95%-CI**	***p***	**HR**	**95%-CI**	***p***	**HR**	**95%-CI**	***p***	**HR**	**95%-CI**	***p***
Age above 55 years	0.358	[0.000; 80.068]	*0.358*	2.370	[0.962; 5.840]	***0.061***	1.803	[0.823; 3.949]	*0.141*	2.618	[1.030; 6.653]	***0.043***
Karnofsky Performance Score	0.840	[0.721; 0.977]	***0.024***	0.932	[0.884; 0.990]	***0.020***	0.932	[0.888; 0.977]	***0.004***	0.962	[0.910; 1.017]	*0.170*
Estrogen receptor positive	0.098	[0.010; 0.946]	***0.045***	0.384	[0.140; 1.049]	***0.062***	0.449	[0.204; 0.985]	***0.046***	0.587	[0.242; 1.426]	*0.239*
Her2/neu receptor rich	3.812	[0.533; 27.256]	*0.182*	1.969	[0.669; 5.798]	*0.219*	1.878	[0.669; 5.274]	*0.232*	0.158	[0.020; 1.270]	***0.083***
Grading G3	1.294	[1.204; 11.623]	*0.872*	1.556	[0.580; 4.173]	*0.380*	1.775	[0.757; 4.163]	*0.187*	3.751	[1.169; 12.044]	***0.026***
SBRT target = bone metastasis	0.019	[0.000; 66.083]	*0.341*	0.225	[0.065; 0.775]	***0.018***	0.172	[0.051; 0.573]	***0.004***	0.117	[0.015; 0.886]	***0.038***
Number of metastases=1	0.019	[0.000; 71.124]	*0.347*	0.371	[0.134; 1.025]	***0.056***	0.491	[0.219; 1.101]	***0.084***	0.916	[0.378; 2.223]	*0.847*
Oligoprogressive disease	11.234	[1.159; 108.877]	***0.037***	1.644	[0.656; 4.118]	*0.289*	1.806	[0.813; 4.011]	*0.146*	1.834	[0.753; 4.467]	*0.182*
BED (α/β = 10)	1.010	[0.971; 1.049]	*0.630*	1.076	[0.997; 1.035]	*0.109*	1.019	[1.001; 1.036]	***0.035***	1.025	[1.000; 1.051]	***0.046***
PTV volume at least 37mL	1.618	[0.226; 11.590]	0.887	0.392	[0.572; 3.383]	*0.466*	1.583	[0.724; 3.460]	*0.250*	3.199	[1.157; 8.847]	***0.025***
												
Chemotherapy within 4 weeks before or after SBRT	14.149	[1.461; 137.050]	**0.022**	1.534	[0.553;4.252]	*0.411*	1.625	[0.679;3.883]	0.275	0.644	[0.214; 1.941]	0.434

*BED, biologically effective dose; CI, confidence interval; DC, distant control; HR, hazard ratio; LC, local control; OS, overall survival; PFS, progression free survival; PTV, planning target volume; SBRT, stereotactic body radiotherapy. The variables BED (α/β = 10) and Karnofsky Performance Score were continuous variables, all other were analyzed as categorical variables. For Her2/neu receptor rich, data was missing for 7 patients and 5 patients had missing data on Grading G3. Bold and italic values indicate p < 0.1. Bold, italic, and underlined values indicate p < 0.05*.

**Table 5:** Multivariate analysis of prognostic factors influencing LC, DC, PFS, and OS.

	**LC**			**DC**			**PFS**			**OS**		
**Factors**	**HR**	**95%-CI**	***p***	**HR**	**95%-CI**	***p***	**HR**	**95%-CI**	***p***	**HR**	**95%-CI**	***p***
Age above 55 years				2.627	[0.970; 7.118]	***0.057***				5.788	[1.077; 31.119]	***0.041***
Karnofsky Performance Score	0.985	[0.774; 1.253]	*0.900*	0.918	[0.850; 0.992]	***0.030***	0.950	[0.891; 1.012]	*0.112*			
Estrogen receptor positive	0.198	[0.009; 4.308]	*0.303*	1.838	[0.524; 6.440]	*0.342*	2.005	[0.688; 5.848]	*0.203*			
Her2/neu receptor rich										1.090	[0.096; 12.437]	*0.944*
Grading G3										1.321	[0.328; 5.320]	*0.695*
SBRT target = bone metastasis				0.272	[0.072; 1.030]	**0.055**	0.022	[0.001; 0.351]	***0.007***	0.849	[0.057; 12.585]	*0.905*
Number of metastases = 1				0.186	[0.055; 0.626]	***0.007***	0.363	[0.152; 0.863]	***0.022***			
Oligoprogressive disease	3.044	[0.210; 44.028]	*0.414*									
BED (α/β = 10)							0.965	[0.923; 1.008]	*0.112*	1.023	[0.966; 1.083]	*0.438*
PTV volume at least 37 mL										3.493	[0.846; 14.425]	***0.084***
Chemotherapy within 4 weeks before or after SBRT	6.904	[0.377; 126.476]	*0.193*									

*BED, biologically effective dose; CI, confidence interval; DC, distant control; HR: hazard ratio; LC, local control; OS, overall survival; PFS, progression free survival; PTV, planning target volume; SBRT, stereotactic body radiotherapy. The variables BED (α/β = 10) and Karnofsky Performance Score were continuous variables, all other were analyzed as categorical variables. For Her2/neu receptor rich, data was missing for 7 patients and 5 patients had missing data on Grading G3. Bold and italic values indicate p < 0.1. Bold, italic, and underlined values indicate p < 0.05*.

### Distant Control

Twenty out of 46 patients (43.5%) were diagnosed with progression distant to the SBRT lesion during follow-up. One and 2 year DC rates were 68.6% and 43.9% (Figure 2B). KPS (HR = 0.932, CI [0.884; 0.990], *p* = 0.020) and bone metastases as the SBRT treating site (HR = 0.225, CI [0.065; 0.775], *p* = 0.018; Figure 3C) appeared to be significant favorable prognostic factors in univariate analysis (Table 4), with the overall number of one metastasis at borderline significance level (HR = 0.371, CI [0.134; 1.025], *p* = 0.056). Patients with higher KPS (HR = 0.918, CI [0.850; 0.992], *p* = 0.030) and a solitary metastasis (HR = 0.186, CI [0.055; 0.626], *p* = 0.007; Figure 3D) were at significantly lower risk of developing distant progression in multivariate analysis (Table 5).

### Progression Free Survival

**Figure 4:**
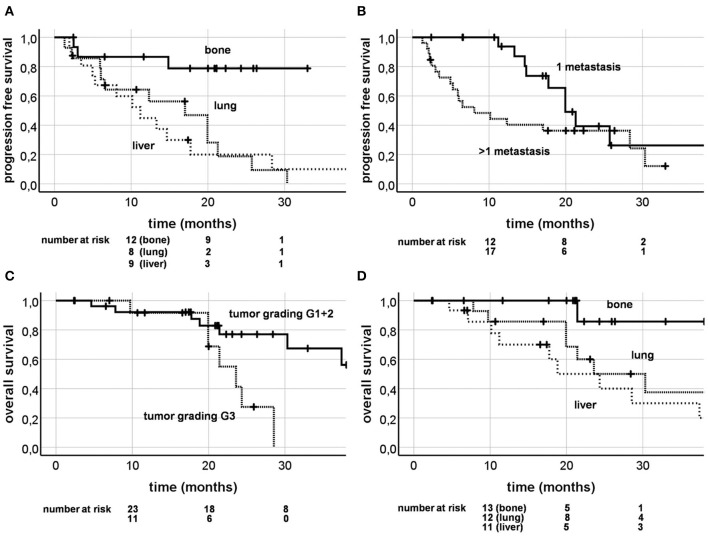
Kaplan-Meier curves; progression free survival depending on the irradiation site (**A**; *p* = 0.004) and present metastases (**B**; *p* = 0.084); overall survival depending on tumor grading (**C**; *p* = 0.026) and on irradiation site (**D**; *p* = 0.038).

During follow-up, 28 progressions or deaths occurred (60.9%). One and 2 year PFS rates were 54.3 and 16.6% (Figure 2C). KPS (HR = 0.932, CI [0.888; 0.977], *p* = 0.004), estrogen receptor positivity (HR = 0.449, CI [0.204; 0.985], *p* = 0.046) and bone metastases as SBRT lesions (HR = 0.172, CI [0.051; 0.573], *p* = 0.004) were shown to be positive prognostic factors in univariate analysis, with single metastasis at borderline significance level (HR = 0.491, CI [0.219; 1.101], *p* = 0.084) and a higher BED as a significant unfavorable factor (HR = 1.019, CI [1.001; 1.036], *p* = 0.035). In multivariate analysis, only bone metastases as SBRT target (HR = 0.022, CI [0.001; 0.351, *p* = 0.007; Figure 4A) and a solitary metastasis (HR = 0.363, CI [0.152; 0.863], *p* = 0.022; Figure 4B) remained as significant favorable factors (Table 5).

### Overall Survival

Twenty-two patients (47.8%) died during follow-up time. One and 2 year OS were 85.4 and 62.1% (Figure 2D). Univariate analysis revealed age over 55 years (HR = 2.618, CI [1.030; 6.653], *p* = 0.043), tumor grading G3 (HR = 3.751, CI [1.169; 12.044], *p* = 0.026; Figure 4C), higher BED (HR = 1.025, CI [1.000; 1.051], *p* = 0.046), and PTV volume ≥37mL (HR = 3.199, CI [1.157; 8.847], *p* = 0.025) as significant unfavorable factors influencing OS. Bony lesions as SBRT target was identified a favorable prognostic factor (HR = 0.117, CI [0.015; 0.886], *p* = 0.038; Figure 4D). In multivariate analysis, only age over 55 years stayed significant (HR = 5.788, CI [1.077; 31.119], *p* = 0.041; Table 5).

## Discussion

In this retrospective study consisting of 46 patients who received ablative SBRT for their 58 extracranial metastases, we sought to describe outcome patterns in comparison to the resulting toxicity and searched for prognostic factors. Our findings resemble the statement concluded in a review by Dorota Kwapisz, that the ideal patients for SBRT in oligometastatic or oligoprogressive breast cancer are young, have a good performance status and a low tumor burden (28) (Tables 4, 5).

In our study, the dominant failure pattern was distant, with 1 and 2 year LC of 92 and 89% vs. DC of 69 and 44%. Table 6 illustrates the most important further studies analyzing SBRT in the treatment of OMD patients. LC at 2 years was shown to be excellent in our study (89%) and comparable to other studies (88–100%; Table 6) (3, 5, 19, 20, 29, 30). In our study, patients with OPD or chemotherapy within 4 weeks before or after SBRT, had significantly less local control rates (Table 4; Figure 3B). This fact has already been described for lung cancer patients treated with SBRT, who showed inferior LC if they had received systemic therapy before (31). This may be due to the changes in tumor biology through systemic treatment, selecting resistant clones (15). Patients with previous systemic therapies might require higher doses to overcome this effect. Furthermore, prior chemotherapy as a negative prognostic factor for local control has also been reported in a large cohort of patients treated with hepatic SBRT (*n* = 452 lesions) (32). Interestingly, 56 breast cancer patients were included in the cohort and also showed inferior LC after the admission of prior chemotherapy. In our study, DC at 2 years (44%) was comparable to the only other study (50%) that analyzed this outcome factor (29). Yet, 2 year PFS and OS in our study were rather low (17% and 62%). This might be due to the fact, that 30% patients had OPD and therefore were more likely to show further disease progression shortly after SBRT. On the other hand, the rather low PFS and OS could also be explained by the high proportion of lung and liver metastases (66%) and consequently lower proportion of bone metastases (33%). Bone metastases are, in contrast to lung and liver metastases, a positive prognostic factor for OS (4), which was also shown in our study (Table 4). Moreover, PFS and OS at 2 years were highest in the study population by David et al. with bone only metastases (65 and 100%) (30) and lowest in the study population by Onal et al. with liver only metastases (8 and 57%) (5).

**Table 6:** Prospective and retrospective studies investigating ablative, stereotactic radiotherapy for oligometastastic breast cancer.

	**Patients, design, characteristics**	**Treated lesions**	**Gy @ isodose**	**Significant prognostic factors in multivariate analysis**	**CTC toxicity**	**2 y. LC**	**2 y. DC**	**2 y. PFS**	**2 y. OS**
Milano et al. (29)	*n =* 40; ≤ 5 mets; KPS ≥70 Prospective pilot study OMD: 100%	*n =* 85 17% bone 22% lung 39% liver 18% lymph node	10 ×5 @ 80%^*^	Negative: GTV (*patient* LC)	III°: *n =* 1 pleural/peri-cardial effusion ≥IV°: 0%^*^	80% (4 y.)	50%	44%	76%
Yoo et al. (19)	*n =* 50; ≤ 5 mets retrospective OMD: 100%	*n =* n/a 100% bone	“median dose 30 Gy (range 20–60)”	Positive: hormone receptor positivity (OS) and single bone metastasis (OS)	n/a	70% (3 y.)	n/a	n/a	85%
Scorsetti et al. (20)	*n =* 33; ≤ 5mets (lung/liver); ECOG ≤ 2 observational study OMD: 100%	*n =* 43 100% lung or liver	3 ×18.75–25 Gy @ 95% 4 ×12 Gy@ 95%	None	I–II°: 18% ≥III°: 0%	90%	n/a	27%	66%
Onal et al. (5)	*n =* 22; ≤ 5 mets retrospective OMD: 100%	*n =* 29 100% liver	3 ×18 Gy @ 90%	None	III°: *n =* 2 (rib fracture, duodenal ulcer) ≥IV°: 0%	88%	n/a	8%	57%
Trovo et al. (3)	*n =* 54; ≤ 5 mets; ECOG ≤ 1; prospective, multicenter phase II trial FDG-PET/CT staging OMD: 100%	*n =* 92 66% bone 25% lymph node 5% liver 4%lung	3 ×10–15 Gy (isodose n/a) 25 ×2,4 Gy IMRT	None	II°: *n =* 2 (pain/fatigue) ≥III°: 0%	97%	n/a	53%	95%
David (30)	*n =* 15; ≤ 3 bone only mets; ECOG ≤ 2 prospective Na-18-F-PET/CT staging OMD: 100%	*n =* 19 100% bone	1 ×20 Gy @ 80%	Not tested	I°: 67% II°: 27% ≥III°: 0%	100%	n/a	65%	100%
Weykamp et al. (present study)	*n =* 46; KPS ≥70 retrospective OMD: 70% ( ≤ 3 mets) OPD: 30% (1 met progressive)	*n =* 58 bone 33% lung 33% liver 33% adrenal 1%	1 ×24–30 @ 90–95% 3 ×15–18 @ 65% 8 ×7.5 @ 80% 10 ×5 @ 90% (bone: 1 ×24 @ 80% or 3 ×9 @ 80%)	Positive: overall present mets ≤ 1 (DC; PFS), KPS (DC); bone metastasis as SBRT target (PFS)Age ≥55 (OS)	I°: 16% II°: 2% ≥III°: 0%	89%	44%	17%	62%

* *not mentioned in the cited paper, “10 ×5 Gy” was obtained from a different citation investigating additional other primary tumors (14)*.

*CTC, Common Terminology Criteria; DC, distant control; ECOG, Eastern Cooperative Oncology Group Performance Status; FDG, fluoro-deoxy-glucose; Gy, Gray; LC, local control; Mets, metastases; n/a, not available; Na-18-F, natriumfluoride-18; OMD, oligometastatic disease; OPD, oligoprogressive disease; OS, overall survival; PET, positron emission tomography; PFS, progression free survival; y, years*.

Significant positive prognostic factors for PFS in multivariate analysis were overall number of metastases (*n* = 1) and bone metastases as the SBRT target (Table 5; Figures 4A,B), the latter was already shown by Yoo et al. (19). Furthermore, patients with one metastastic lesion were already reported to have a more favorable outcome (29). A higher BED as a prognostic factor for superior OS has been described by Hong et al. (33) in 361 patients (16% breast cancer) treated with SBRT for their oligometastases. Surprisingly, in our study, univariate analysis described a higher BED as a negative prognostic factor for PFS and OS (Table 4). This is probably caused by the fact that bone metastases had a better outcome with less radiation dose. Accordingly, BED did not remain a significant factor in multivariate analysis.

Furthermore, patients who received SBRT for their bone metastases showed a significantly longer OS in our univariate analysis. However, this did not persist in multivariate analysis, after adjusting for age. As Milano et al. had described before, breast cancer patients with bone metastases are more likely to be of young age (4). A PTV volume of at least 37 mL was a negative prognostic factor for OS in univariate analysis, which might reflect a higher tumor burden. Similar results were shown by Milano et al. describing a higher GTV negatively influencing LC (Table 6) (29). As expected, tumor grading G3 had a negative impact on OS in univariate analysis (Table 4), reflecting a more aggressive disease.

Interestingly, the KPS was a significant positive prognostic factor for LC, DC and PFS (Table 4) and stayed significant for DC in multivariate analysis (Table 5). The above mentioned two prospective studies on SBRT for oligometastatic breast cancer by Milano et al. and David et al. used a certain performance index threshold for inclusion into the respective study (29, 30). Similarly, our cohort consists of patients with a relatively high KPS, with a median of 90% and a range of 70–100%.

Recently, Murano et al. reported that SBRT in oligometastatic breast cancer patients resulted in an increase or even new appearance of polyfunctional CD4+ and CD8+ T-cells against breast cancer antigens (34). Since SBRT is thought to promote immunogenic cell death, it may also lead to a treatment benefit not only in local control of the irradiated lesion, but also in distant control (35, 36). This might be caused by the so called “abscopal effect,” which describes a “response at a distance from the irradiated volume” (37). However, breast cancer is so far not considered a typical immunogenic cancer (38). Nonetheless, especially triple negative or Her2/neu rich breast cancer seems to show a high proportion of tumor infiltrating immune cells (39, 40). Results of a recently published Phase-III trial could show a prolonged disease free survival in metastasized breast cancer patients when adding Atezolizumab to Nab-Paclitaxel chemotherapy (41). SBRT is thought to be less affected by a high mutation load, which leads to the interesting concept to use SBRT to postpone a change of systemic therapy (15). For patients with oligoprogressive lung, renal cell or prostate cancer, several recent studies have already investigated the role of additional local treatment to the progressive lesions (31, 42–44). To date, there has been no dedicated study published for breast cancer patients with OPD which goes beyond the plane description of the progression pattern (45).

To our knowledge, our study is the first in the field to also include and analyze oligoprogressive patients with widespread metastatic disease. Patients with OMD had a maximum of three present metastases, compared to a maximum of one progressive lesion in patients with OPD, pointing toward a more cautious and stricter definition of limited metastatic disease in case of oligoprogressive disease. Patients with OPD showed an inferior, yet satisfying local control after SBRT, which may be due to a higher mutation burden in these patients. A dose escalation concept could be investigated to overcome this suspected higher radioresistance. Interestingly, DC, PFS and OS were not significantly different in OPD patients, which would have been expected otherwise due to their worse prognosis from the outset (45). Moreover, though nearly a third of our study population consists of OPD patients, outcome was still comparable to other studies in the field only including oligometasatic breast cancer patients (Table 6). Consequently, SBRT should also be investigated in OPD patients in further studies. In future, SBRT for a few progressive metastases in widespread metastastic disease could help to postpone a change in systemic therapy and hence help to change a fatal cancer state into a chronic disease.

The main limitation of this study is its retrospective design. Unlike other, prospective studies, patients did not receive fluoro-deoxy-glucose (FDG) positron emission tomography (PET) or natirumfluoride-18 (Na-18-F) PET scan as initial staging (3, 30). Hence, those patients with less favorable outcome in our study might have had more metastases than detected during contrast enhanced CT scan staging.

The above mentioned survey of Canadian medical oncologists revealed a high proportion of doubt considering SBRT for oligometastastic breast cancer (18). Studies investigating high dose chemotherapy for metastatic breast cancer were mainly conducted when Weichselbaum and Hellman developed their theory of OMD in the 1990s (1, 46). Compared to standard dose chemotherapy, little benefit could be achieved with this approach, at the cost of higher toxicity (46). These experiences may have led to the perception, that therapy escalation for (oligo)metastatic breast cancer patients in general is rather harmful. A certain amount of skepticism due to the lack of phase III studies for SBRT in oligometastatic breast cancer is understandable. Moreover, the SABR-COMET trial, which can be considered one of the most important studies addressing the concept of ablative therapies in OMD in general, revealed a risk of CTC V° toxicities (4.5%; each *n* = 1 radiation pneumonitis, pulmonary abscess and subdural hemorrhage after surgery due to the SBRT). To our best knowledge, no grade IV or higher toxicity was described in the aforementioned studies on SBRT in breast cancer patients with OMD (Table 6), with only very few grade III toxicities. In consistence with other studies in the field, our study demonstrates SBRT as a well-tolerated ablative therapy. The growing acceptance of OMD and even OPD as a disease concept is reflected by our recently increasing treatment sessions (Figure 1).

Based on the promising results of the SABR-COMET trial, future prospective studies need to focus on OMD and OPD in breast cancer to evaluate the benefit of SBRT added to systemic treatment. The German OLIGOMA study will address this particular topic in near future and includes breast cancer patients with up to five metastases (47). The NRG BR002 study was commenced in 2016 and investigates additional SBRT or surgery in breast cancer patients with OMD compared to standard therapy alone (48). Nonetheless, robust data on the expected benefit of local ablative therapy in breast cancer patients with OMD and OPD will take years to be available. Until then, SBRT in oligometastastic or oligoprogressive breast cancer patients should be strongly considered as a highly effective treatment option to eradicate local metastases with only mildest toxicity. As shown in our retrospective study with an equal proportion of the three most common metastatic organs (bone, liver, and lung), SBRT provides excellent local control and is safe outside a clinical trial. Moreover, in times of more and more expensive systemic therapy options, SBRT offers a cost effective treatment approach compared to other local ablative treatments (49–51).

## Conclusion

Extracranial SBRT in breast cancer patients with OMD or OPD is well-tolerated with excellent LC. The ideal patient is of young age, has only one metastasis and reaches an excellent performance score. The increase in utilization since 2017 points toward a growing acceptance of SBRT for OMD and OPD in breast cancer. Future trials are highly needed to consolidate the role of local ablative treatment in both oligometastatic and oligoprogressive breast cancer patients.

## Data Availability Statement

The datasets generated for this study are available on request to the corresponding author.

## Ethics Statement

The studies involving human participants were reviewed and approved by Ethics Committee of Heidelberg University Faculty of Medicine. Written informed consent for participation was not required for this study in accordance with the national legislation and the institutional requirements.

## Author Contributions

FW carried out the data collection, performed the statistical analysis, and drafted the manuscript. LK, KS, TF, PH, SA, SM, and SW helped with data collection as well as figure and table preparation. FW, LK, KS, SA, and JH-R were involved in patient treatment. TD and AS contributed the gynecological knowledge of the manuscript and were involved in pre-radiotherapy treatment. JH-R and JD participated in the study design and helped to draft the manuscript. All the authors were responsible for data interpretation, participated in manuscript revisions, and approved the final manuscript.

## Conflict of Interest

The authors declare that the research was conducted in the absence of any commercial or financial relationships that could be construed as a potential conflict of interest.

## References

[1] Weichselbaum R Hellman S. Oligometastases. J Clin Oncol. (1995) 13:8–10. 10.1200/JCO.1995.13.1.8 7799047

[2] Hanrahan EO Broglio KR Buzdar AU Theriault RL Valero V Cristofanilli M. Combined-modality treatment for isolated recurrences of breast carcinoma: update on 30 years of experience at the University of Texas MD Anderson Cancer Center and assessment of prognostic factors. Cancer Interdisc Int J Am Cancer Soc. (2005) 104:1158–71. 10.1002/cncr.21305 16047352

[3] Trovo M Furlan C Polesel J Fiorica F Arcangeli S Giaj-Levra N. Radical radiation therapy for oligometastatic breast cancer: results of a prospective phase II trial. Radiother Oncol. (2018) 126:177–80. 10.1016/j.radonc.2017.08.032 28943046

[4] Milano MT Katz AW Zhang H Huggins CF Aujla KS Okunieff P. Oligometastatic breast cancer treated with hypofractionated stereotactic radiotherapy: some patients survive longer than a decade. Radiother Oncol. (2019) 131:45–51. 10.1016/j.radonc.2018.11.022 30773186

[5] Onal C Guler OC Yildirim BA. Treatment outcomes of breast cancer liver metastasis treated with stereotactic body radiotherapy. Breast. (2018) 42:150–6. 10.1016/j.breast.2018.09.006 30296648

[6] Bartlett EK Simmons KD Wachtel H Roses RE Fraker DL Kelz RR. The rise in metastasectomy across cancer types over the past decade. Cancer. (2015) 121:747–57. 10.1002/cncr.29134 25377689

[7] Hong JC Salama JK. The expanding role of stereotactic body radiation therapy in oligometastatic solid tumors: what do we know and where are we going? Cancer Treat Rev. (2017) 52:22–32. 10.1016/j.ctrv.2016.11.003 27886588

[8] Rieber J Streblow J Uhlmann L Flentje M Duma M Ernst I. Stereotactic body radiotherapy (SBRT) for medically inoperable lung metastases—a pooled analysis of the German working group “stereotactic radiotherapy”. Lung Cancer. (2016) 97:51–8. 10.1016/j.lungcan.2016.04.012 27237028

[9] Sterzing F Brunner TB Ernst I Baus WW Greve B Herfarth K. Stereotactic body radiotherapy for liver tumors: principles and practical guidelines of the DEGRO Working Group on Stereotactic Radiotherapy. Strahlenther Onkol. (2014) 190:872–81. 10.1007/s00066-014-0714-1 25091267

[10] Andratschke N Alheid H Allgäuer M Becker G Blanck O Boda-Heggemann J. The SBRT database initiative of the German Society for Radiation Oncology (DEGRO): patterns of care and outcome analysis of stereotactic body radiotherapy (SBRT) for liver oligometastases in 474 patients with 623 metastases. BMC Cancer. (2018) 18:283. 10.1186/s12885-018-4191-2 29534687 PMC5851117

[11] Gomez DR Tang C Zhang J Blumenschein GR Hernandez M Lee JJ Local consolidative therapy vs. maintenance therapy or observation for patients with oligometastatic non–small-cell lung cancer: long-term results of a multi-institutional, phase ii, randomized study. J Clin Oncol. (2019) 37:1558–65. 10.1200/JCO.19.00201 31067138 PMC6599408

[12] Palma DA Olson R Harrow S Gaede S Louie AV Haasbeek C. Stereotactic ablative radiotherapy versus standard of care palliative treatment in patients with oligometastatic cancers (SABR-COMET): a randomised, phase 2, open-label trial. Lancet. (2019) 393:2051–8. 10.1016/S0140-6736(18)32487-5 30982687

[13] Wong AC Watson SP Pitroda SP Son CH Das LC Stack ME. Clinical and molecular markers of long-term survival after oligometastasis-directed stereotactic body radiotherapy (SBRT). Cancer. (2016) 122:2242–50. 10.1002/cncr.30058 27206146

[14] Milano MT Katz AW Muhs AG Philip A Buchholz DJ Schell MC A prospective pilot study of curative-intent stereotactic body radiation therapy in patients with 5 or fewer oligometastatic lesions. Cancer Interdisc Int J Am Cancer Soc. (2008) 112:650–8. 10.1002/cncr.23209 18072260

[15] Patel P Palma D McDonald F Tree A The Dandelion dilemma revisited for oligoprogression: treat the whole lawn or weed selectively? Clin Oncol. (2019) 31:824–33. 10.1016/j.clon.2019.05.015 PMC6880295 31182289

[16] Dias M Antunes A Campainha S Conde S Barroso A. Prognostic impact of M descriptors of the 8th edition of TNM classification of lung cancer. J Thorac Dis. (2017) 9:685. 10.21037/jtd.2017.03.106 28449476 PMC5394072

[17] Wang M Chen H Wu K Ding A Zhang M Zhang P. Evaluation of the prognostic stage in the 8th edition of the American Joint Committee on Cancer in locally advanced breast cancer: an analysis based on SEER 18 database. Breast. (2018) 37:56–63. 10.1016/j.breast.2017.10.011 29100045

[18] Donovan E Dhesy-Thind S Mukherjee S Kucharczyk M Swaminath A. Attitudes and beliefs toward the use of stereotactic body radiotherapy in oligometastatic breast cancer: a commentary on a survey of Canadian Medical Oncologists. Breast J. (2019) 25:1222–4. 10.1111/tbj.13435 31264272

[19] Yoo GS Yu JI Park W Huh SJ Choi DH. Prognostic factors in breast cancer with extracranial oligometastases and the appropriate role of radiation therapy. Radiat Oncol J. (2015) 33:301–9. 10.3857/roj.2015.33.4.301 26756030 PMC4707213

[20] Scorsetti M Franceschini D De Rose F Comito T Villa E Iftode C. Stereotactic body radiation therapy: a promising chance for oligometastatic breast cancer. Breast. (2016) 26:11–7. 10.1016/j.breast.2015.12.002 27017237

[21] Scorsetti M Franceschini D De Rose F Comito T Franzese C Masci G. The role of SBRT in oligometastatic patients with liver metastases from breast cancer. Rep Pract Oncol Radiother. (2017) 22:163–9. 10.1016/j.rpor.2016.07.008 28490988 PMC5411907

[22] Timmerman R McGarry R Yiannoutsos C Papiez L Tudor K DeLuca J. Excessive toxicity when treating central tumors in a phase II study of stereotactic body radiation therapy for medically inoperable early-stage lung cancer. J Clin Oncol. (2006) 24:4833–9. 10.1200/JCO.2006.07.5937 17050868

[23] Timmerman R Paulus R Galvin J Michalski J Straube W Bradley J. Stereotactic body radiation therapy for inoperable early stage lung cancer. JAMA. (2010) 303:1070–6. 10.1001/jama.2010.261 20233825 PMC2907644

[24] Park C Papiez L Zhang S Story M Timmerman RD. Universal survival curve and single fraction equivalent dose: useful tools in understanding potency of ablative radiotherapy. Int J Radiat Oncol Biol Phys. (2008) 70:847–52. 10.1016/j.ijrobp.2007.10.059 18262098

[25] Wöckel A Stüber T editors. S3-Leitlinie “Interdisziplinäre Früherkennung, Diagnose, Therapie und Nachsorge des Mammakarzinoms”. Forum. Basel: Springer (2019).

[26] Liedtke C Jackisch C Thill M Thomssen C Müller V Janni W. AGO recommendations for the diagnosis and treatment of patients with early breast cancer: update 2018. Breast Care. (2018) 13:196–208. 10.1159/000489329 28785186 PMC5527197

[27] Thill M Liedtke C Müller V Janni W Schmidt M Committee AB. AGO recommendations for the diagnosis and treatment of patients with advanced and metastatic breast cancer: update 2018. Breast Care. (2018) 13:209–15. 10.1159/000489331 30069182 PMC6062660

[28] Kwapisz D. Oligometastatic breast cancer. Breast Cancer. (2019) 26:138–46. 10.1007/s12282-018-0921-1 30324552

[29] Milano MT Zhang H Metcalfe SK Muhs AG Okunieff P. Oligometastatic breast cancer treated with curative-intent stereotactic body radiation therapy. Breast Cancer Res Treat. (2009) 115:601–8. 10.1007/s10549-008-0157-4 18719992

[30] David S. Stereotactic ablative body radiotherapy (SABR) for bone only oligometastatic breast cancer: a prospective clinical trial. Breast. (2019) 49:55–62. 10.1016/j.breast.2019.10.016 31734589 PMC7375645

[31] Lara TM Helou J Poon I Sahgal A Chung HT Chu W Multisite stereotactic body radiotherapy for metastatic non-small-cell lung cancer: delaying the need to start or change systemic therapy? Lung Cancer. (2018) 124:219–26. 10.1016/j.lungcan.2018.08.005 30268464

[32] Klement RJ Guckenberger M Alheid H Allgäuer M Becker G Blanck O. Stereotactic body radiotherapy for oligo-metastatic liver disease – influence of pre-treatment chemotherapy and histology on local tumor control. Radiother Oncol. (2017) 123:227–33. 10.1016/j.radonc.2017.01.013 28274491

[33] Hong JC Ayala-Peacock DN Lee J Blackstock AW Okunieff P Sung MW. Classification for long-term survival in oligometastatic patients treated with ablative radiotherapy: a multi-institutional pooled analysis. PloS ONE. (2018) 13:e0195149. 10.1371/journal.pone.0195149 29649281 PMC5896920

[34] Muraro E Furlan C Avanzo M Martorelli D Comaro E Rizzo A. Local high-dose radiotherapy induces systemic immunomodulating effects of potential therapeutic relevance in oligometastatic breast cancer. Front Immunol. (2017) 8:1476. 10.3389/fimmu.2017.01476 29163540 PMC5681493

[35] Bhalla N Brooker R Brada M. Combining immunotherapy and radiotherapy in lung cancer. J Thorac Dis. (2018) 10(Suppl. 13):S1447. 10.21037/jtd.2018.05.107 29951296 PMC5994496

[36] Ko EC Raben D Formenti SC. The integration of radiotherapy with immunotherapy for the treatment of non–small cell lung cancer. Clin Cancer Res. (2018) 24:5792–806. 10.1158/1078-0432.CCR-17-3620 29945993

[37] Dagoglu N Karaman S Caglar HB Oral EN. Abscopal effect of radiotherapy in the immunotherapy era: systematic review of reported cases. Cureus. (2019) 11:e4103. 10.7759/cureus.4103 31057997 PMC6476623

[38] Miglietta F Griguolo G Guarneri V Dieci MV. Programmed cell death ligand 1 in breast cancer: technical aspects, prognostic implications, and predictive value. Oncologist. (2019) 24:e1055–69. 10.1634/theoncologist.2019-0197 31444294 PMC6853089

[39] Savas P Salgado R Denkert C Sotiriou C Darcy PK Smyth MJ. Clinical relevance of host immunity in breast cancer: from TILs to the clinic. Nat Rev Clin Oncol. (2016) 13:228. 10.1038/nrclinonc.2015.215 26667975

[40] Stanton SE Adams S Disis ML. Variation in the incidence and magnitude of tumor-infiltrating lymphocytes in breast cancer subtypes: a systematic review. JAMA Oncol. (2016) 2:1354–60. 10.1001/jamaoncol.2016.1061 27355489

[41] Schmid P Adams S Rugo HS Schneeweiss A Barrios CH Iwata H. Atezolizumab and nab-paclitaxel in advanced triple-negative breast cancer. N Engl J Med. (2018) 379:2108–21. 10.1056/NEJMoa1809615 30345906

[42] Qiu B Liang Y Li Q Liu G Wang F Chen Z. Local therapy for oligoprogressive disease in patients with advanced stage non–small-cell lung cancer harboring epidermal growth factor receptor mutation. Clin Lung Cancer. (2017) 18:e369–73. 10.1016/j.cllc.2017.04.002 28465010

[43] Triggiani L Alongi F Buglione M Detti B Santoni R Bruni A. Efficacy of stereotactic body radiotherapy in oligorecurrent and in oligoprogressive prostate cancer: new evidence from a multicentric study. Br J Cancer. (2017) 116:1520. 10.1038/bjc.2017.103 28449007 PMC5518848

[44] Santini D Ratta R Pantano F De Lisi D Maruzzo M Galli L. Outcome of oligoprogressing metastatic renal cell carcinoma patients treated with locoregional therapy: a multicenter retrospective analysis. Oncotarget. (2017) 8:100708. 10.18632/oncotarget.20022 29246014 PMC5725056

[45] Kelly P Ma Z Baidas S Moroose R Shah N Dagan R. Patterns of progression in metastatic estrogen receptor positive breast cancer: an argument for local therapy. Int J Breast Cancer. (2017) 2017:1367159. 10.1155/2017/1367159 29147583 PMC5632870

[46] Gianni L. High-dose chemotherapy for breast cancer: any use for it? Ann Oncol. (2002) 13:650–2. 10.1093/annonc/mdf232 12075732

[47] Krug D Fabian A Pyschny F Blanck O Dellas K Maass N Strahlentherapie bei Patientinnen mit oligometastasiertem Mammakarzinom. Der Gynäkol. (2019) 52:918–26. Available online at: https://www.springermedizin.de/mammakarzinom/mammakarzinom/strahlentherapie-bei-patientinnen-mit-oligometastasiertem-mammak/17122456

[48] Chmura SJ Winter KA Al-Hallaq HA Borges VF Jaskowiak NT Matuszak M NRG-BR002: a phase IIR/III trial of standard of care therapy with or without stereotactic body radiotherapy (SBRT) and/or surgical ablation for newly oligometastatic breast cancer (NCT02364557). J Clin Oncol. (2019) 37(Suppl. 15):TPS1117 10.1200/JCO.2019.37.15_suppl.TPS1117

[49] Possanzini M Greco C. Stereotactic radiotherapy in metastatic breast cancer. Breast. (2018) 41:57–66. 10.1016/j.breast.2018.06.011 30007269

[50] Sher DJ Wee JO Punglia RS. Cost-effectiveness analysis of stereotactic body radiotherapy and radiofrequency ablation for medically inoperable, early-stage Non-small cell lung cancer. Int J Radiat Oncol. (2011) 81:e767–74. 10.1016/j.ijrobp.2010.10.074 21300476

[51] Bijlani A Aguzzi G Schaal D Romanelli P. Stereotactic radiosurgery and stereotactic body radiation therapy cost-effectiveness results. Front Oncol. (2013) 3:77. 10.3389/fonc.2013.00077 23580234 PMC3619246

